# Robotic assisted laparoscopic prostatectomy in a patient with prostate cancer and complex urinary tract malformation

**DOI:** 10.1016/j.eucr.2021.101613

**Published:** 2021-03-17

**Authors:** Mohamed Abd-Alazeez, Sidath H. Liyanage, Juliet Campbell, Maria Guzha, Mohamed Saad, Peter Acher

**Affiliations:** Southend University Hospital, Mid and South Essex NHS Foundation Trust, UK

**Keywords:** Robot, Prostate cancer, Ectopic ureter, Supernumerary kidney

## Abstract

We present a case of prostate cancer with abnormal renal and ureteric anatomy who underwent robotic assisted laparoscopic prostatectomy.

This is a 59-year-old European patient who presented with lower urinary tract symptoms (LUTS) and pelvic pain. Investigations revealed prostate cancer as well as a supernumerary right kidney and an atrophic horseshoe left kidney draining into the left seminal vesicle. He was managed with robotic assisted laparoscopic prostatectomy (RALP) using a modified technique.

Selective pre-operative investigations and patient counselling led to proper operative planning and good surgical technique and outcome.

## Introduction

Supernumerary kidney is a rare upper urinary tract malformation in which an accessory kidney is present in addition to the normal two native kidneys. Theories of development suggest either splitting of the metanephric blastema or formation of an additional one. This results into either partially or completely duplicated ureters. The supernumerary kidney may be either separate from the normal one or fused with loose areolar tissue. It is reported more commonly on the left side.^1^ This condition is usually asymptomatic; incidental diagnosis typically occurs upon radiological studies done for other reasons. Those who develop symptoms usually present around the age of 35.

Associated anomalies including horseshoe kidney, ectopic ureter and ureteral atresia, are common.^2^

## Case presentation

A 59-year-old man presented in primary care with urinary symptoms and vague pelvic pain. His GP arranged for CT scan of the abdomen and pelvis and a PSA blood test. He was then referred to our centre following the finding of a raised PSA (9.3mcg/L). The patient suffered from mixed mild LUTS with an IPSS of 7 and QoL of 3. There was no history of UTI or visible haematuria. His past medical history included left inguinal hernia repair associated with left simple orchidectomy for atrophic testicle, at 11 years of age.

Abdominal examination revealed the scar related to left inguinal hernia repair. Digital rectal examination revealed a firm right prostate base.

The patient then underwent a multi-parametric MRI (mpMRI) scan of the prostate (T2W, DW and Dynamic Contrast Enhanced sequences) prior to transperineal prostate biopsy.

**Fig. 1:**
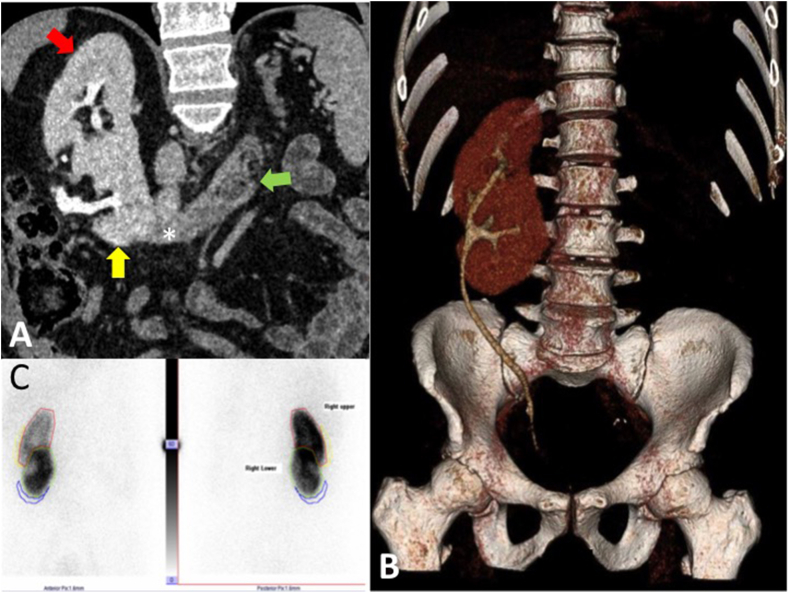
(A) Coronal oblique contrast enhanced CT reconstruction in the excretory phase shows two kidneys in the right lumbar region. The upper kidney (red arrow) has an anterior orientation whilst the lower partially fused kidney (yellow arrow) has an anterolateral orientation. The atrophic left kidney with poor cortical enhancement and no contrast excretion is seen on the left (green arrow) and is joined to the supernumerary right kidney by a fibrous parenchymal bridge (asterisk). (B) 3 D reconstruction of contrast enhanced CT excretory phase showing right supernumerary kidney with lower kidney renal pelvis rotated anterolateral and both ureters joining together and taking a normal course to insert into the urinary bladder. The atrophic left horseshoe kidney shows poor parenchymal enhancement and no contrast excretion and therefore is not seen on this image. (C) DMSA scan shows all the activity in the right supernumerary kidney with no activity in the atrophic horseshoe left kidney. (For interpretation of the references to colour in this figure legend, the reader is referred to the Web version of this article.)

**Fig. 2:**
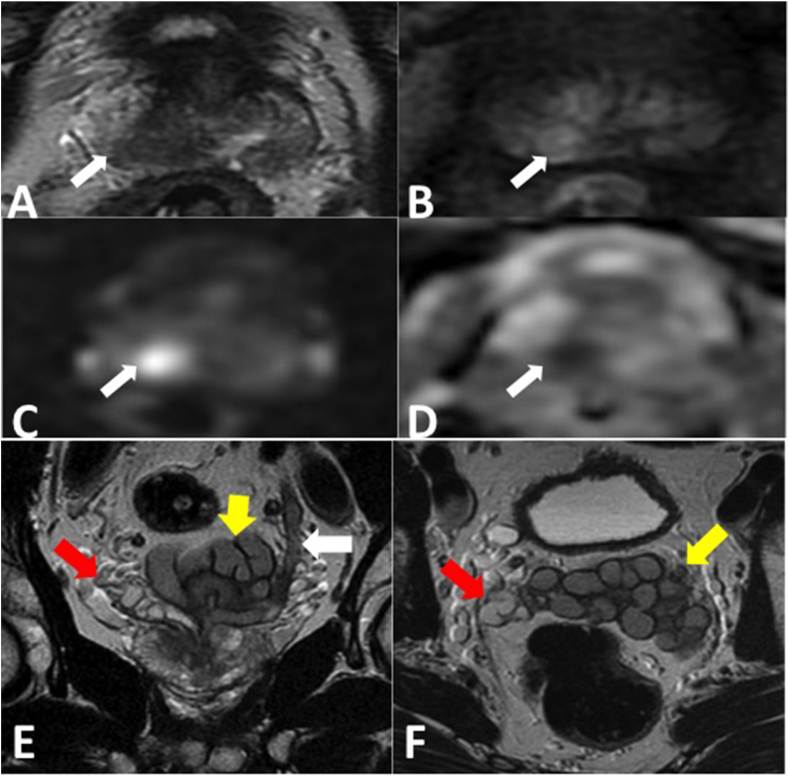
**A-D**: Multi-parametric MRI. Axial T2w (A), dynamic contrast enhanced (B) DWI (C) and ADC map (D) show a hypointense circumscribed area of low T2 signal intensity in the right medial basal peripheral zone (arrow) which displays focal early asymmetrical enhancement and is high signal on the DWI with corresponding low signal intensity on the ADC. This is scored as a PI-RADS 4/5 lesion as the size of the abnormality is less than 1.5 cm, stage T2. Targeted biopsy revealed Gleason 3 + 4. **E& F:** Coronal T2w(E) and axial T2w(F) of the prostate and seminal vesicles show the ectopic insertion of the left ureter (white arrow) into the left seminal vesicle. The left seminal vesicle (yellow arrow) is asymmetrically enlarged compared to the normal sized right seminal vesicle (red arrow). (For interpretation of the references to colour in this figure legend, the reader is referred to the Web version of this article.)

His CT and mpMR imaging together with biopsy findings, were then discussed in our multidisciplinary team (MDT) meeting. CT urogram (Fig. 1) showed a horseshoe kidney with atrophic left part and a supernumerary right kidney. The ureter of the atrophic left horseshoe kidney was draining into the ipsilateral seminal vesicle whereas both the right horseshoe and the supernumerary right kidneys drained via a single ureter into the urinary bladder. MpMR images (Fig. 2) showed a 26 cc prostate with a PI-RADS 4/5 lesion at the right posterior peripheral zone at the base. They also showed an abnormally enlarged left seminal vesicle. His prostate biopsies confirmed right side Gleason 3 + 4 in a significant number of cores. Consensus from MDT meeting was that the patient should be offered active treatment options for his clinically significant prostate cancer. The patient opted for surgery and so a DMSA scan was arranged in order to assess the split function of the kidneys. The left horseshoe kidney showed no function; RALP was planned with intraoperative ligation of the left distal ureter.

As per BAUS guidelines, during the COVID-19 pandemic, the patient was started on hormone treatment for prostate cancer for nearly 100 days whilst waiting to have surgery.

**Fig. 3:**
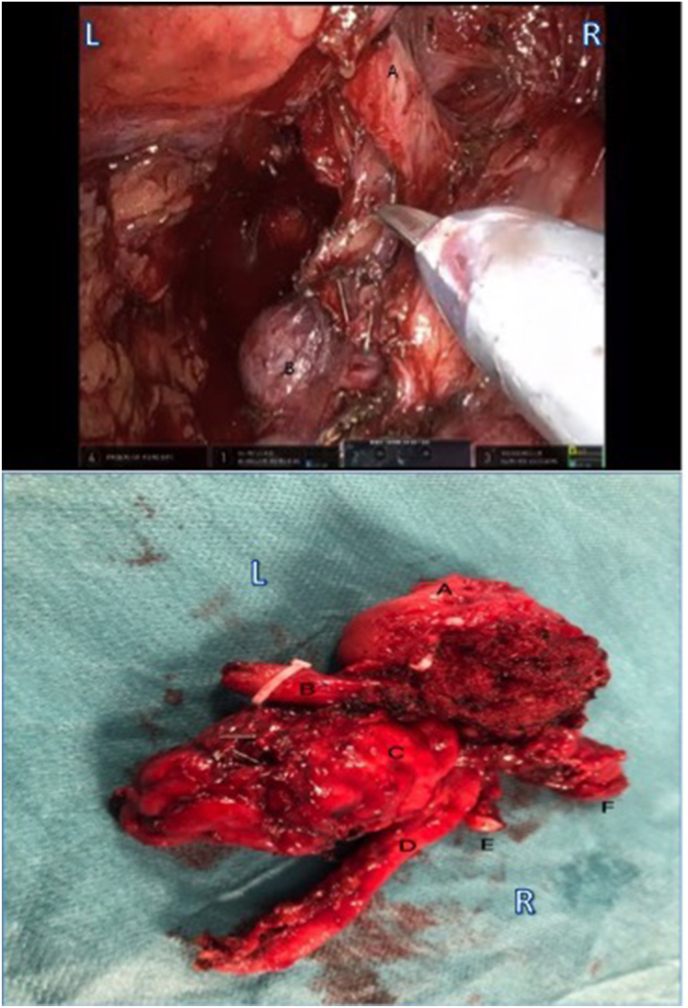
**Left:** Intraoperative screenshot during posterior approach part of the procedure. A; left ureter, B; left seminal vesicle. **Right**: Postoperative prostatectomy specimen. A; prostate, B; left ureter, C; left seminal vesicle, D; left vas deferens, E; right vas deferens, F; right seminal vesicle.

At the time of robotic prostatectomy, the surgeon adopted the posterior approach in order to identify the structures at the bladder base more easily. Both seminal vesicles were identified and dissected. The vasa were also revealed and cut. The left ureter was encountered near its opening into the left SV (Fig. 3). This was cross-clamped and cut. The operation was then completed using the anterior approach with right side wide excision and left intrafascial nerve sparing. Console time was 2:30 hours and no complications occurred. Estimated blood loss was 100 mls.

Postoperatively, the patient's condition was stable and urinary output was good. Estimated GFR (kidney function) remained normal. The patient was deemed medically fit for discharge the following morning. Postoperative histology revealed pT3bNxMxR0 Gleason 3 + 4 prostate adenocarcinoma and confirmed the histology of the cut ureter (Fig. 3). The patient was reviewed 4 weeks postoperatively and had no loin pain. Follow up CT urogram showed no hydronephrosis in the atrophic left horseshoe kidney whereas there was normal uptake and excretion by the right kidneys. He was fully continent at 8 weeks with undetectable PSA.

## Discussion

We believe that our case demonstrated multiple rare features with the presence of a right (instead of the usual left) supernumerary kidney, horseshoe kidney (with atrophic left one), ectopic insertion of the left ureter and the presence of prostate cancer. To the best of our knowledge, this is the first case in the literature to be reported with these features for a male patient who was managed with robotic prostatectomy for his prostate cancer disease.

Supernumerary kidney is different from the known case of crossed fused ectopia in which the crossed kidney retains its native ureteric opening on the contralateral side. Another differential diagnosis is “duplex kidney” involving a bifid renal pelvis that may be partial or complete to form two separate ureters. However, in supernumerary kidney there is one renal pelvis and each of the normal kidney and supernumerary one has its own capsule, renal artery and vein.

Ruling out any function in the atrophic left kidney through DMSA scan was crucial before planning for surgery. The surgeon was prepared to look for the left ureter, and safely ligate it.

Gonzalvo Pérez et al. presented a case of supernumerary kidney in a female patient with ectopic vaginal ureteral opening and horseshoe kidney.^3^ Unal et al. were also first to report a case of supernumerary kidney associated with coarctation of the aorta.^4^

Matsumoto et al. reported a similar case, where a 44y old patient presented with macroscopic haematuria. Investigations revealed an ipsilateral atrophic kidney with an ectopic ureter draining into the ipsilateral seminal vesicle. A diagnosis of high-risk prostate cancer was also made. Laparoscopic nephro-ureterectomy and open radical prostatectomy were performed.^5^

## Conclusions

We believe that stepwise management in our case was essential in order to obtain the optimal investigations for satisfactory counselling of the patient along with meaningful discussion in our Urology MDT meeting. Having detailed clinical and radiological information contributed to a successful surgical outcome with no intra- or post-operative complications.

Robotic surgery clearly helps provide minimally invasive treatment in these cases with excellent details for such complex anatomy.
